# *Escherichia coli* and *Klebsiella pneumoniae* Carbapenemase in Long-term Care Facility, Illinois, USA

**DOI:** 10.3201/eid1506.081735

**Published:** 2009-06

**Authors:** Marcella McGuinn, Ronald C. Hershow, William M. Janda

**Affiliations:** University of Illinois, Chicago, Illinois, USA

**Keywords:** Escherichia coli, Klebsiella pneumoniae carbapenemase, bacteria, long-term care facility, Illinois, USA, letter

**To the Editor:** *Escherichia coli* harboring *Klebsiella pneumoniae* carbapenemases (KPCs) are now rarely being reported. Worldwide, KPC-2 has been detected in Israel and the People’s Republic of China (*1*,*2*). Within the United States, carbapenem-resistant *E. coli* carrying *bla*_KPC_ has been isolated in New Jersey (*3*) and Cleveland, Ohio (*4*), and 7 carbapenem-resistant *E. coli* isolates were obtained from 3 different hospitals in Brooklyn, New York (*5*). Urban et al. (*6*) recently reported 9 KPC-2 and KPC-3 carbapenemases in urinary *E. coli* isolates from 7 long-term care facilities. We report such an isolate from a resident of a long-term care facility.

This case involved a 68-year-old female resident of a long-term care facility in Centralia, Illinois, who had multiple chronic medical problems, including cerebral palsy, a seizure disorder, and recurrent urinary tract infections. A urine culture grew >10^5^ CFU/mL of *E. coli* susceptible to amikacin, gentamicin, tobramycin, piperacillin/tazobactam, trimethoprim/sulfamethoxazole, imipenem, and nitrofurantoin. Tigecycline susceptibility was not determined. Trimethoprim/sulfamethoxazole therapy was initiated. Follow-up urine culture almost 3 weeks later again grew >10^5^ CFU/mL of *E. coli*, now susceptible to amikacin, gentamicin, tobramycin, nitrofurantoin, and tigecycline. The isolate was resistant to imipenem and meropenem. A modified Hodge test demonstrated production of a carbapenemase (*7*), and the *bla*_KPC_ gene was detected by PCR at the Centers for Disease Control and Prevention (CDC). The patient was treated with a 10-day course of nitrofurantoin, 100 mg by gastrostomy tube 2× per day. Chart review indicated that contact precautions were instituted only after discovery of the second *E. coli* isolate.

Seventeen days later, a repeat urine culture grew >10^5^ CFU/mL of *K. pneumoniae* susceptible only to amikacin, gentamicin, tobramycin, and tigecycline. No treatment was given. Follow-up urine culture grew >10^5^ CFU/mL of *K. pneumoniae* again with a similar resistance pattern. The modified Hodge test result was positive (*7*) and was confirmed as *bla*_KPC_ positive by PCR at CDC. The resident was transferred to an acute care facility for further evaluation and was treated with amikacin. At completion of therapy, a repeat urine culture was negative for organisms.

Our case, like that of Urban et al. (*6*), involved a urinary isolate from a resident of a long-term care facility. As increasing numbers of resistant gram-negative rods colonize such patients, the patients may acquire a bacterium carrying a KPC plasmid conferring broad-spectrum resistance as described in our patient. These plasmids may then be laterally transferred to other gram-negatives, which may have occurred in this case.

Our case underscores the gravity of the evolutionary process of emergent, multidrug–resistant enterobacteriaceae. Even though *E. coli* strains that harbor carbapenemase genes are not ubiquitous, additional therapeutic interventions are needed to prevent the spread of these bacteria, which are likely to infect increasing numbers of patients.
